# Short-term outcomes of Oxford unicompartmental knee arthroplasty with coronal subluxation of the knee: a retrospective case–control study

**DOI:** 10.1186/s10195-022-00626-x

**Published:** 2022-01-21

**Authors:** Gang Xi, Hao-hao Wang, Hao Li, Min Zhang

**Affiliations:** Department of Orthopedics Surgery, The Second Affiliated Hospital of Shanxi Medical University, Taiyuan, 382 Wuyi Road, Xinghualing District, Taiyuan, 030001 China

**Keywords:** Knee, Coronal tibiofemoral subluxation, Unicompartmental knee arthroplasty, Osteoarthritis, Treatment outcome

## Abstract

**Background:**

The goal of this study was to assess short-term outcomes in single compartment osteoarthritis patients associated with the coronal tibiofemoral subluxation (CTFS) of the knee joint after Oxford unicompartmental knee arthroplasty (OUKA), and to establish the potential impact of the degree of CTFS on operative outcomes.

**Methods:**

Data pertaining to 183 patients with medial compartment osteoarthritis that underwent OUKA treatment between February 2016 and June 2019 were retrospectively analyzed. The presence and degree of severity of CTFS were assessed using preoperative weight-bearing anteroposterior X-ray images of the knee. Patients were stratified into three subgroups based upon the observed degree of subluxation: a normal group, a mild subluxation group (CTFS < 0.5 cm), and a severe subluxation group (CTFS ≥ 0.5 cm). Anterior and posterior X-ray examination of the knee was conducted at the time of most recent follow-up for each patient to assess the degree of CTFS correction following OUKA. Clinical function was assessed using Oxford knee score (OKS) and Hospital for Special Surgery score (HSS) values, while pain was rated using visual-analog scale (VAS) scores. The mechanical femoral tibial angle (mFTA), range of motion (ROM), and complication rates in these three groups were additionally compared.

**Results:**

The average follow-up duration for patients in this study was 24.1 months (range: 17–32 months). There were no significant differences in patient age, sex, body mass index (BMI), follow-up duration, mFTA, ROM, OKS, HSS, or VAS scores among these three groups (*P* > 0.05). After surgery, OKS and HSS scores declined significantly, but no differences in these scores were observed among groups (*P* > 0.05). Of these patients, 135 (73.8%) were satisfied with the operation, of whom 80 (43.7%) were very satisfied. There were no significant differences in ROM or VAS scores among groups (*P* > 0.05). The degree of CTFS for patients in the mild and severe subluxation groups was significantly improved following OUKA relative to preoperative values such that the degree of postoperative CTFS did not differ significantly among these groups (*P* > 0.05). Postoperative mFTA was also significantly improved in these three patient subgroups (*P* < 0.05). No patients experienced operative complications over the follow-up period.

**Conclusions:**

OUKA can successfully improve clinical symptoms in patients with single compartmental osteoarthritis. Moreover, OUKA can effectively correct CTFS of the knee in these patients, and the degree of preoperative CTFS has no impact on surgical efficacy.

**Level of evidence:**

III.

## Introduction

Oxford unicompartmental knee arthroplasty (OUKA) is an effective approach to treating single-compartmental knee osteoarthritis, and the implementation of this procedure has increased by 30% in recent years. Optimal patient selection is essential to ensure the success of the OUKA procedure [1]. However, controversy remains regarding the most appropriate indications and contraindications for such treatment [2], particularly with respect to the results of imaging evaluations. One potential contraindication for OUKA is tibiofemoral subluxation in patients with univentricular arthritis, although this remains disputed and has not been studied in detail in prior studies. As such, there is currently a dearth of evidence available to guide orthopedic clinicians in the effective identification of feasible surgical approaches [3].

Traditional preoperative imaging analyses typically necessitate that anteroposterior X-ray films be taken in a weight-bearing state to detect the narrowing or disappearance of the joint space of the medial compartment and changes in bone-to-bone contact [4, 5]. X-ray films of the weight-bearing lateral position and 90° flexion lateral position have revealed that the wear site is limited to the front and middle of the medial tibial platform, whereas the articular cartilage behind the medial tibial platform remains intact [6–8]. These analyses, however, fail to account for the potential effects of the coronal subluxation of the knee on OUKA surgical outcomes. Such coronal subluxation is a common radiological finding in knee arthritis patients, and primarily manifests as the dislocation of the tibia relative to the femur in the coronal plane [9, 10]. While the mechanistic basis for this phenotype is poorly understood, it may be linked to severe wear to the cartilage and to ligament relaxation surrounding the knee joint [11, 12] Whether coronal subluxation is a contraindication for OUKA remains a matter of active debate, with some studies having suggested that tibiofemoral subluxation be considered an exclusion criterion for OUKA, whereas others suggest that an OUKA approach may still be applicable for these patients provided the subluxation is “correctable” based upon the results of preoperative pressure imaging [13, 14]. A few prior studies have also explored the effects of differing degrees of coronal subluxation of the knee on single condylar replacement outcomes.

There is currently no general consensus regarding the effects of tibial femoral coronal subluxation on single condylar replacement outcomes. As such, we conducted the present retrospective study to assess single condylar replacement outcomes in single knee osteoarthritis as a function of the degree of subluxation of the tibiofemoral joint, in an effort to guide future clinical practice. For these analyses, osteoarthritis patients were separated into three subgroups based upon the degree of preoperative coronal tibiofemoral subluxation (CTFS), as measured via X-ray imaging. The two primary goals of this study were as follows: (1) to assess improvements in the degree of CTFS following OUKA and (2) to establish the impact of differing degrees of preoperative CTFS on OUKA surgical outcomes.

## Materials and methods

### Study design

This retrospective study was conducted at the Department of Orthopedics in the Second Affiliated Hospital of Shanxi Medical University. The protocol for the study has been approved by the Ethics Committee of the Second Affiliated Hospital of Shanxi Medical University and performed in a manner consistent with the Helsinki Declaration [15]. Written informed consent to participate was obtained from all patients.

### Inclusion and exclusion criteria

Patients eligible for inclusion in this study were individuals who were: (1) diagnosed with knee osteoarthritis and underwent unilateral OUKA at a single hospital between February 2016 and June 2019; (2) implanted with a third-generation Oxford mobile platform prosthesis; (3) exhibited a preoperative CTFS ≤ 1 cm; and (4) had undergone follow-up for ≥ 2 years.

Patients were excluded from this study if they: (1) had a history of prior knee joint surgery; (2) exhibited anterior cruciate ligament (ACL) dysfunction that was detected intraoperatively and underwent simultaneous ACL reconstruction; or (3) were lost to follow-up or had incomplete follow-up data.

In total, 221 patients underwent OUKA treatment for medial single-compartment osteoarthritis over this study period. Of these patients, 183 (62 male, 121 female) met with study inclusion criteria, with an average age of 66.1 years. Clinical data (demographic and functional evaluation data) and radiological parameters (CTFS value and mFTA) for all patients were recorded.

### Surgical methods

The same surgeon treated all patients, all of whom were implanted with an Oxford unicondylar prosthesis. Following anesthetization, patients were placed in the supine position. This limb was placed in a unicondylar leg frame such that the hip joint was flexed 30° with slight abduction while the lower leg drooped naturally, allowing the passive range of motion (ROM) of the knee to reach 110°. A tourniquet was used to disrupt blood flow to the affected limbs.

This surgical procedure was conducted via a medial patellar approach, with the knee joint bent 90°. A 6–7 cm incision was made at the medial edge of the patella extending from above the joint capsule to the tibial tubercle at the distal end. The skin, subcutaneous tissue, and joint capsule were cut in sequence. An Oxford bone spoon of proper thickness was then selected based upon the medial joint space and articular cartilage wear. A vertical osteotomy was then performed on the intercondylar crest of the tibia close to the inner side of the ACL and directed towards the anterior superior iliac spine. After carefully protecting the medial collateral ligament with a Z-shaped retractor, a horizontal osteotomy was then performed, and the osteotomy block was removed and compared with a corresponding test model to determine the sizing of the tibial prosthesis. After the knee joint was flexed 45°, the femoral condyle was fully exposed and a femoral cavity positioning rod was inserted in a retrograde manner, after which the femoral drilling guide was installed. Drill holes along the guide were made with 4 mm and 6 mm electric drills. The femoral posterior condyle osteotomy plate was placed in the femoral condyle positioning hole to adhere to the femoral condyle osteotomy, after which the grinding bolt was placed in the femoral positioning hole, and the distal femur was ground with the grinding drill.

The unicondylar prosthesis was then installed, the flexion–extension gap was repeatedly tested to ensure there was no impact or dislocation. The joint capsule was then washed repeatedly, the joint capsule, subcutaneous tissue, and skin were sutured under sufficient hemostasis, and sterile dressings and elastic bandages were applied to compress the wound.

### Postoperative treatment

Immediately after patients had recovered from anesthetization, ankle pumping and straight leg lifting training were initiated. On day 1 postoperatively, some patients were able to walk with the aid of a walker. Oral anticoagulants were routinely administered to prevent complications such as deep venous thrombosis of the lower limbs. After discharge, patients were instructed to exercise actively, such that the ROM of the knee joint generally extended to 0–100° within 1 week. After 2 weeks, stitches were removed and full weight-bearing walking exercises were initiated.

### Result evaluations

The evaluation process was simultaneously performed by three observers who assessed clinical and imaging parameters. Each relevant measurement or assessment value was performed twice by each of these observers, with the average value then being taken for analysis. The intraclass correlation coefficient (ICC) was used to evaluate the interobserver reliability and retest reliability. If the reliability coefficient was less than 0.4, the reliability was considered poor, whereas if it was greater than 0.75, the reliability was considered to be high.

### Evaluation of clinical function

All clinical information for these patients was collected by a research assistant using a predesigned case report form at 6 weeks, 3 months, 6 months, 12 months, and every year thereafter postoperatively. Outpatient follow-up was conducted using paper questionnaires or via telephone. Primary clinical measurement indices for these patients included the OKS and HSS scores, the former of which has been confirmed to be a reliable evaluation tool following OUKA surgery and the latter of which offers insight regarding changes in the status of the knee joint in knee osteoarthritis patients before and after surgical treatment. Clinical outcomes were described in terms of the percentage of maximum possible improvement (PMPI), rather than by using a predetermined value of the minimum clinically important difference (MCID) as this was considered to be limited by the ceiling effect. A cut-off value of 30% was used to differentiate between the number of patients who were satisfied (PMPI > 30%) and dissatisfied (PMPI < 30%) with their surgical procedure as determined based upon HSS score results. This cut-off value has previously been used in the joint replacement literature to indicate significant and functional improvement [16, 17]. The basic principle of this method is that it sets a higher standard than does MCID, increasing the number of patients with unsatisfactory results and allowing comparisons between these patients and those with satisfactory results. During follow-up, patients graded their operative satisfaction as follows: disappointed, dissatisfied, neutral, satisfied, or very satisfied. ROM values and complication rates in each patient group were additionally recorded, and VAS scores were used to rate the degree of knee pain before and after surgery.

### OKS scores

OKS scores consist of 12 items, each of which was initially scored from 1–5 points, with one corresponding to the slightest and five corresponding to the most serious outcome for that item. Total scores range from 12 points (best) to 60 points (worst). Many scholars eventually suggested that these scores be adjusted to range from 0–4, with a maximum possible score of 48 [18]. Accordingly, the latest revised OKS scoring system rates each item from 0–4, with total possible scores ranging from 0 (worst) to 48 (best).

### HSS scores

The HSS knee score is a scoring system proposed in 1976 by the American Second Hospital of Special Surgery to evaluate knee joints before and after surgical procedures. This scale assesses six key aspects: pain, function, ROM, muscle strength, knee flexion deformity, and knee instability. The total possible HSS score is 100 points (pain: 30 points; function: 22 points; ROM: 18 points; muscle strength: 10 points; knee flexion deformity: 10 points; knee instability: 10 points). The results are divided into four grades: excellent (> 85 points), good (70–84 points), moderate (60–69 points), and poor (< 59 points) [19].

### VAS scores

A 10 cm horizontal line was drawn on a sheet of paper, with a 0 at one end of the line indicating no pain, and a 10 at the other end indicating severe pain. Patients were directed to indicate the region on this scale that matched their degree of pain. The distance from 0 to the marked position was then measured to determine the VAS score for a given patient [20].

### Imaging parameters

In radiographic images, the same source to image distance (SID) before and after operation was measured, and the SID value was set to 80 cm. Subluxation of the tibia relative to the femur was measured on preoperative anterior and posterior X-ray images of the knee joint in a weight-bearing position. Based on the degree of subluxation, patients were separated into three groups: normal, mild subluxation, and severe subluxation groups. At their most recent follow-up, anterior and posterior X-ray examinations of these patients were again performed to measure the degree of correction of such subluxation by the OUKA procedure. In addition, mFTA was measured using the full-length films of both lower limbs to judge lower limb alignment before and after surgery.

### CTFS measurement

**Fig. 1:**
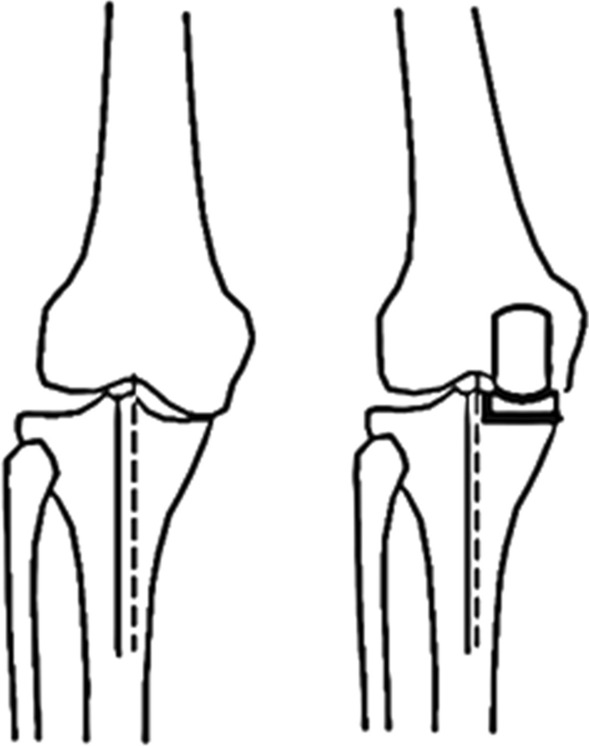
The proximal tibial axis (solid line) is drawn through the midpoint of the tibial crest and the midpoint of the tibial shaft about 120 mm from the tibial articular surface, with a parallel dotted line representing the proximal tibial axis (dotted line) being drawn through the apex of the femoral intercondylar fossa. The distance between these two lines is the CTFS value

Many approaches to measuring CTFS have been described to date. As the diseased knee joint often lacks clear anatomical landmarks owing to osteophyte hyperplasia, hypertrophy, or other defects, such measurements can be challenging. For this study, CTFS measurements were made as per a protocol published by Nam et al. [14], which avoids the influence of osteophytes while also reducing the influence of the slight rotation of X-ray film (Fig. 1).

### Complications

Complications such as incision problems, neurovascular injury, periprosthetic infection, deep vein thrombosis (DVT), pulmonary embolism (PE), knee joint stiffness, prosthesis loosening, gasket dislocation, and osteoarthritis progression in the lateral compartment were analyzed for all patients.

### Statistical analysis

SPSS 26.0 (IBM, IL, USA) was used for all statistical analyses. Quantitative data are given as means ± standard deviation, and were compared using paired *t*-tests, independent-sample *t*-tests, and one-way ANOVAs with Bonferroni correction, as appropriate. Categorical data were given as absolute frequencies (*n*), and were compared via Pearson’s Chi-squared test with a two-sided *α*-value of 0.05, and *P* < 0.05 as the threshold for significance.

## Results

### Follow-up

A total of 156 patients (85%) received regular outpatient follow-up, while 27 patients (15%) were followed up via telephone because they could not accept outpatient follow-up for specific reasons. The mean follow-up time for patients in this study was 24.1 months (24.2 ± 4.2, 23.8 ± 5.0, and 25.0 ± 5.0 months in the normal, mild subluxation, and severe subluxation groups, respectively), with no significant differences among groups (*P* = 0.466) (Table 1). Patients in all three groups exhibited clear improvements in osteoarthritis symptoms at the end of the study period.

**Table 1:** Patients baseline data

Parameters	All patients (*n* = 183)	Normal group (*n* = 68)	Mild subluxation group (*n* = 85)	Severe subluxation group (*n* = 30)	*P*-value
Age, years	66.1 ± 7.4	66.8 ± 6.9	65.7 ± 7.6	65.5 ± 7.8	0.592
Gender (male/female)	62/121	25/43	31/54	6/24	0.214
BMI, kg/m^2^	26.0 ± 4.0	26.1 ± 3.9	25.8 ± 3.9	26.6 ± 4.3	0.609
Mechanical FTA, °	179.7 ± 2.4	179.5 ± 2.1	179.6 ± 2.5	180.7 ± 2.6	0.052
Preoperative ROM, °	107.2 ± 10.6	105.9 ± 9.8	107.7 ± 10.7	108.7 ± 11.7	0.395
Preoperative OKS Score	36.9 ± 4.4	37.1 ± 4.3	36.8 ± 5.0	36.7 ± 2.8	0.922
Preoperative HSS Score	59.4 ± 10.7	59.4 ± 10.5	59.9 ± 10.1	57.7 ± 12.9	0.622
Preoperative VAS Score	6.4 ± 0.9	6.3 ± 0.9	6.4 ± 0.9	6.7 ± 0.9	0.111

### General results

The normal group included 68 patients (25 male, 43 female), with an average age of 66.8 ± 6.9 years and an average BMI of 26.8 ± 4.3 kg/m^2^. The mild subluxation group included 85 patients (31 male, 54 female), with an average age of 65.7 ± 7.6 years and an average BMI of 26.8 ± 4.3 kg/m^2^. The severe subluxation group included 30 patients (6 male, 24 female), with an average age of 65.5 ± 7.8 years and an average BMI of 26.6 ± 4.3 kg/m^2^. Baseline patient findings are presented in Table 1. There were no differences among groups with respect to patient age, gender, BMI, mFTA, preoperative ROM, preoperative OKS scores, preoperative HSS scores, or preoperative VAS scores (*P* > 0.05).

### Clinical results

**Fig. 2:**
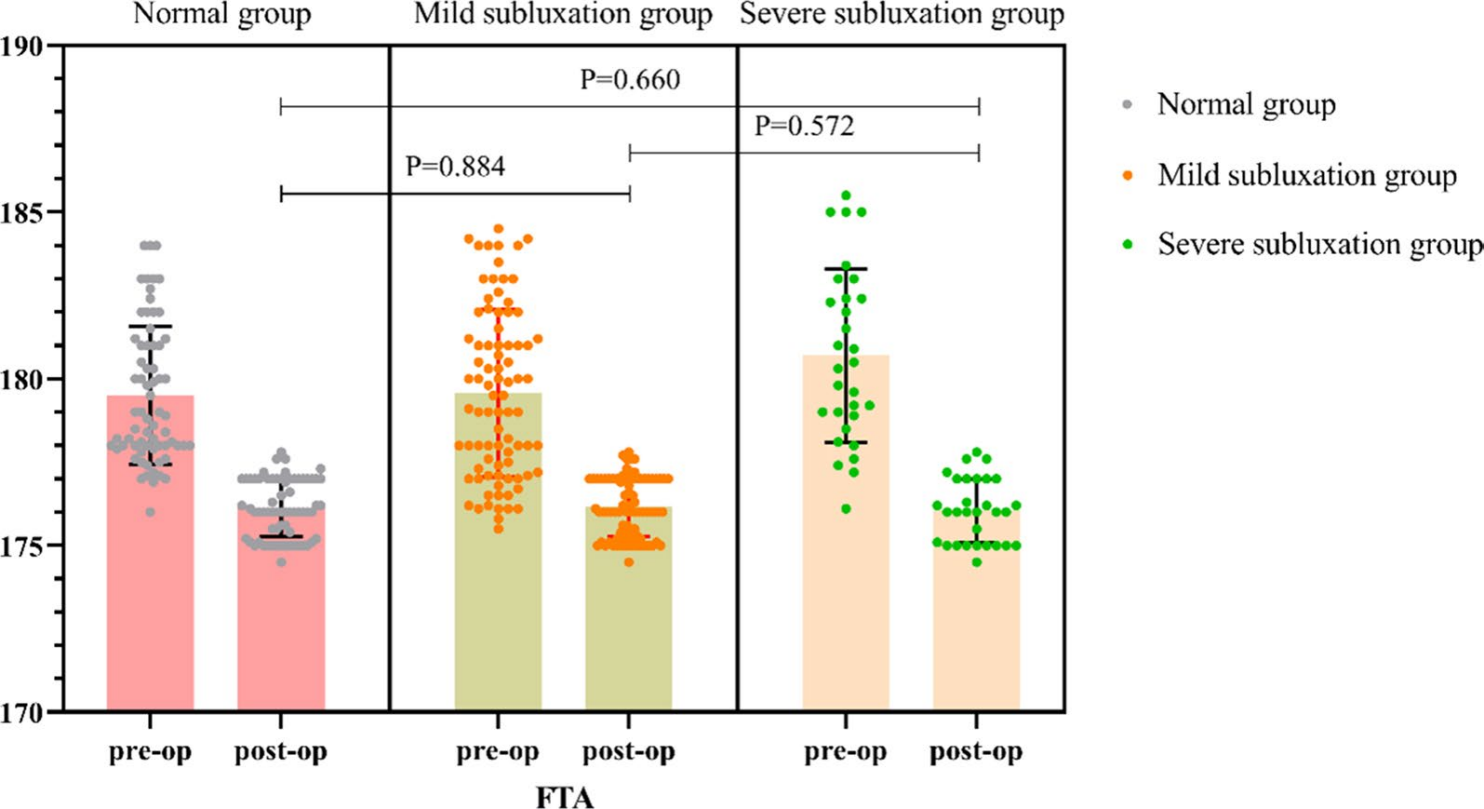
**A** Preoperative anterior and posterior X-ray images of the knee exhibited “bone on bone” contact in the medial compartment, with a measured CTFS of 0.62 cm; **B** Anterior and posterior X-ray images of the knee after OUKA revealed satisfactory prosthesis positioning, with a measured CTFS of 0 cm

The overall follow-up time for these patients was 24.1 ± 4.7 months, including 24.1 ± 4.2 months in the normal group, 23.8 ± 5.0 months in the mild subluxation group, and 25.0 ± 5.0 months in the severe subluxation group. Obvious improvements in clinical function after surgery were evident for patients in all three groups (Fig. 2). As of most recent follow-up, the ROM of patients in the normal, mild subluxation, and severe subluxation groups was 121.1 ± 10.7°, 120.8 ± 10.4°, and 120.6 ± 10.3°, with no significant differences among groups (*P* = 0.974). OKS scores in the normal group improved from 37.1 ± 4.3 to 19.7 ± 3.5, while those in the mild subluxation group improved from 36.8 ± 5.0 to 19.5 ± 3.7, and those in the severe subluxation group improved from 36.7 ± 2.8 to 18.2 ± 3.9, with no significant differences among these groups (*P* = 0.153). The HSS score for patients in the mild subluxation group rose from 59.9 ± 10.1 to 83.9 ± 7.3, while that of patients in the severe subluxation group increased from 57.7 ± 12.9 to 86.2 ± 7.8, with no significant difference between these groups (*P* = 0.293). Of these 183 patients, 135 (73.8%) were satisfied with the results of their procedure, including 80 who were very satisfied. Osteoarthritis-related pain also markedly improved in all three groups following OUKA treatment, with no differences in postoperative pain scores among the normal (1.8 ± 0.8), mild subluxation (1.8 ± 0.7), and severe subluxation (1.7 ± 0.8) groups (*P* = 0.555) (Table 2).

**Table 2:** Postoperative clinical results in patient subluxation subgroups

Parameters	All patients (*n* = 183)	Normal group (*n* = 68)	Mild subluxation group (*n* = 85)	Severe subluxation group (*n* = 30)	*P*-value
Postoperative ROM, °	120.9 ± 10.5	121.1 ± 10.7	120.8 ± 10.4	120.6 ± 10.3	0.974
Postoperative OKS Score	19.4 ± 3.7	19.7 ± 3.5	19.5 ± 3.7	18.2 ± 3.9	0.153
Postoperative HSS Score	84.7 ± 7.2	85.0 ± 7.0	83.9 ± 7.3	86.2 ± 7.8	0.293
Postoperative VAS Score	1.8 ± 0.8	1.8 ± 0.8	1.8 ± 0.7	1.7 ± 0.8	0.555

### Radiological parameters

**Fig. 3:**
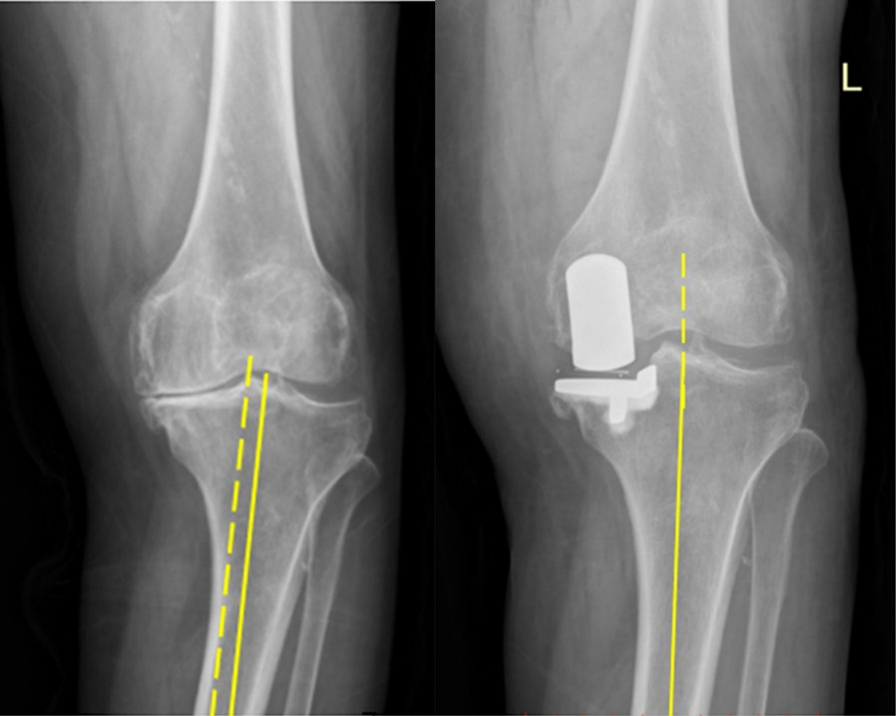
Comparison of CTFS between the mild subluxation group and the severe subluxation group

**Fig. 4:**
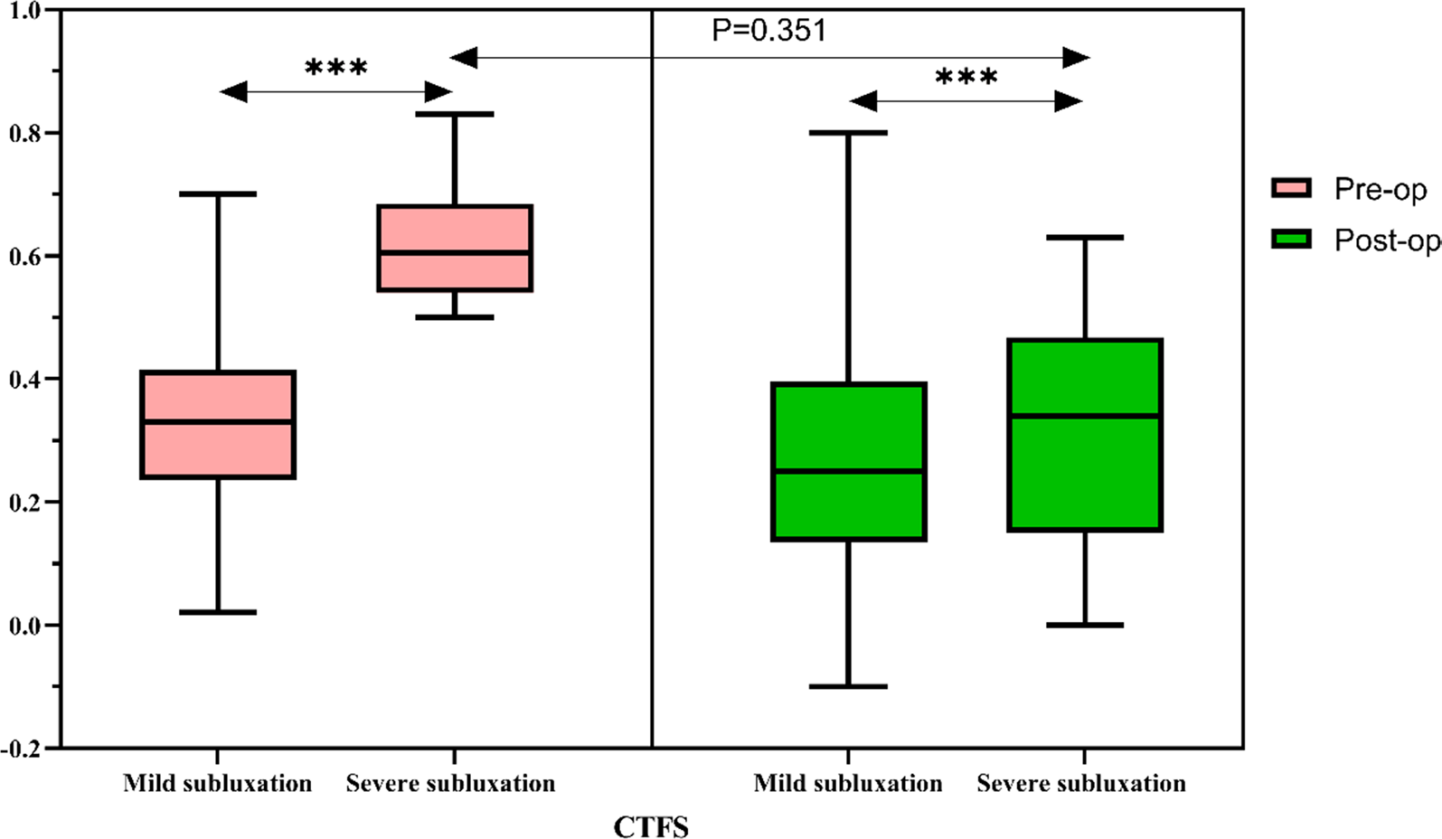
Comparison of FTA in the three subluxation subgroups

As of the most recent follow-up, the average CTFS in the mild subluxation group was 0.27 ± 0.27 cm, which was a significant improvement over the preoperative measure (0.31 ± 0.13 cm) (*P* < 0.05), and the average CTFS in the severe subluxation group was 0.32 ± 0.18 cm, which was similarly significantly improved compared with the preoperative value (0.63 ± 0.10 cm) (*P* < 0.05), with no significant differences between groups (*P* = 0.351) (Fig. 3). The FTA of the three groups was improved compared with that before operation (*P* < 0.05), although there was no significant difference between the groups (*P* = 0.851) (Fig. 4).

### Complications

No patients in this study cohort experienced significant vascular or neurological complications during the follow-up period, nor did any experience prosthetic loosening, periprosthetic infection, or gasket complications such as dislocation, or lateral compartment osteoarthritis progression.

## Discussion

### The mechanistic basis for CTFS

In patients with knee osteoarthritis, the coronal subluxation of the knee is often overlooked, in part because it has rarely been discussed in prior studies. However, such CTFS does exist and has the potential to impact the prognosis of patients undergoing knee arthroplasty [21]. While the exact causes of CTFS remain uncertain, potential mechanisms include inherent knee soft tissue relaxation and changes in subchondral bone mass. Khamaisy et al. [11] determined that CTFS primarily occurs during the early stages of osteoarthritis and is unrelated to osteoarthritic severity. This may be because inherent soft tissue relaxation around the knee occurs during the early stages of osteoarthritis, driving CTFS development. As osteoarthritis continues to progress, this soft tissue may stiffen, potentially preventing further subluxation. Other researchers have instead posited that the quality of subchondral bone plays a major role in the occurrence of CTFS [12]. Premature or excessive subchondral bone hardening in the context of osteoarthritis may lead to the conversion of loads borne by the joint into lateral shear force on the hardened surface, ultimately inducing CTFS. When such subchondral bone hardening is insufficient, the load borne by the join can further wear away this relatively weak surface, such that these patients may exhibit deep wear on the tibial side without signs of lateral subluxation [22, 23]. We did not explore the specific causes of CTFS in the present study as some patients exhibit findings inconsistent with either model, such that they exhibit neither the relaxation of soft tissue around the knee joint nor any obvious subchondral bone quality abnormalities. However, as many patients exhibit the relaxation of soft tissue around the knee in our experience, we speculate that this may be a major driver of CTFS development. CTFS is particularly important in the context of the OUKA operation, as the purpose of OUKA is to restore the physiological tension of the medial collateral ligament, and the impact of ligament relaxation on OUKA warrants further exploration and verification.

### The impact of CTFS on OUKA operation outcomes

One important reason to measure and study CTFS values is that they can supplement the indications for OUKA and guide efforts to predict patient outcomes after this procedure. In preoperative X-ray evaluations for patients scheduled to undergo OUKA, clinicians primarily focus on osteophytes, the joint space, subchondral sclerosis, and bone defects [24, 25]. CTFS is rarely mentioned as an evaluation index [26], despite the fact that these CTFS values can be of substantial practical significance. On the one hand, the emergence of CTFS may be indicative of serious local cartilage wear. Siriwanarangsun et al. [27] found that the cartilage degeneration of the medial surface of the lateral femoral condyle and the tibial crest was particularly pronounced in magnetic resonance imaging (MRI) scans of patients with CTFS. Berger et al. [28] further determined that the coronal subluxation of the knee can lead to the impact of the tibial crest on the femoral condyle, thereby accelerating the progression of knee osteoarthritis. In the present study, we also confirmed the existence of such a bone-on-bone impact phenomenon. In patients with a high degree of CTFS, cartilage defects caused by impact at the medial edge of the lateral femoral condyle were also observed, although these defects were usually in non-weight-bearing areas and so should not affect the OUKA procedure. CTFS may additionally impact surgical efficacy in patients with knee osteoarthritis. Vainionpää et al. [29] reported for the first time in 1981 that coronal subluxation of the knee may adversely impact high tibial osteotomy, with many other authors having since confirmed their findings [30–32]. However, how CTFS impacts prosthesis survival rates following knee arthroplasty remains controversial. Some studies have demonstrated that CTFS can result in the excess concentration of stress at the medial edge of the tibial prosthesis after replacement, leading to negative outcomes including prosthesis loosening and accelerated gasket wear [14, 26, 33]. Collier et al. [34] retrospectively assessed 245 cases of single condylar replacement performed by the same surgeon between 1988 and 1997, and found that there was no significant correlation between the revision rate of single condylar replacement and the preoperative CTFS value. In a study conducted by Nam et al. [14], surgical results from 235 patients undergoing medial UKA and 39 patients undergoing lateral UKA were retrospectively analyzed, revealing no significant differences in CTFS values between UKA patients and healthy controls. In our study, we found that the surgical efficacy of OUKA was positive in the normal and combined CTFS groups. These similar results may be attributable to the correction of CTFS after OUKA in our two subluxation patient groups. Additionally, there was no evidence of prosthesis loosening or gasket wear as a concentration of postoperative stress concentrated at the inner edge of the tibial prosthesis, which may be related to the short-term follow-up of this study.

### CTFS as a predictor of ACL function

Another reason why CTFS warrants further study is that it can aid in the verification of ACL functional status during the OUKA procedure. Vanionpää et al. [29] reported that knee joints with CTFS > 12 mm often exhibit forward instability of the knee joint. Springer et al. [35] assessed the relationship between ACL functional status and CTFS in knee varus osteoarthritis patients, and found that 64% of patients with ACL dysfunction exhibited CTFS > 6 mm, whereas just 1 out of 41 patients exhibited ACL dysfunction among patients with CTFS < 6 mm. These results thus suggest a strong correlation between ACL dysfunction and increased CTFS. The ACL serves as a de facto “lifeline” for the OUKA procedure, and good ACL function is thus a prerequisite for this surgical approach. The primary approach to preoperative ACL evaluation in patients scheduled to undergo OUKA recommended by Oxford at present is to assess the worn portion in the standard 90° lateral position [36, 37]. These results also suggest that CTFS can be used as an auxiliary means of assessing the functional status of the ACL in these patients to further guide appropriate treatment efforts. For knee osteoarthritis patients with a large preoperative CTFS, knee stability and ACL function must be carefully evaluated, and the OUKA operation should not be performed where either of these processes is compromised.

## Strengths and limitations

One major strength of this study is that CTFS was applied to the preoperative evaluation system for OUKA, complementing a vacancy in this field and providing better guidelines for the development of significant clinical work. However, this study is subject to certain limitations. For one, this was a retrospective case-control study, and is thus susceptible to selection bias. Additionally, inconsistencies in the projection angle and body positioning of different patients and for the same patient at pre- and postoperative time points are likely to have introduced measurement errors. Finally, the overall sample size of this study was relatively small and the follow-up duration was relatively short. The long-term implications of these findings necessitate additional larger studies conducted over an extended period to confirm and expand upon our results.

## Conclusion

OUKA represents an effective approach to treating knee osteoarthritis, additionally enabling the correction of preoperative coronal subluxation of the knee joint. Differences in the degree of CTFS should not be considered as a contraindication for OUKA in otherwise eligible patients.

## Data Availability

The datasets used and/or analyzed during the current study are available from the corresponding author on reasonable request.

## Abbreviations

CTFS: Coronal tibiofemoral subluxation
OUKA: Oxford unicompartmental knee arthroplasty
OKS: Oxford knee score
HSS: The Hospital for Special Surgery
VAS: Visual-analog scale
mFTA: Mechanical femoral tibial angle
ROM: Range of motion
BMI: Body mass index
